# Tuberous Sclerosis: A Case Report and Review of the Literature

**DOI:** 10.7759/cureus.12481

**Published:** 2021-01-04

**Authors:** Klenam Dzefi-Tettey, Emmanuel K Edzie, Philip Gorleku, Albert D Piersson, Obed Cudjoe

**Affiliations:** 1 Radiology, Korle-Bu Teaching Hospital, Accra, GHA; 2 Medical Imaging, University of Cape Coast, Cape Coast, GHA; 3 Diagnostic Imaging, University of Cape Coast, Cape Coast, GHA; 4 Anatomical Sciences, University of Cape Coast School of Medical Sciences, Cape Coast, GHA

**Keywords:** tuberous sclerosis, angiofibromas, subependymal giant cell astrocytoma, renal angiomyolipomas, seizures

## Abstract

Tuberous sclerosis (TS) is a rare genetic disorder of autosomal-dominant inheritance. Mutations on either of the two genes Tuberous Sclerosis Complex 1 (TSC1) or Tuberous Sclerosis Complex 2 (TSC2) play a role and result in hamartomas involving many organs, like the brain, heart, kidneys, skin, lungs, and liver. This case report is about a four-year-old boy with facial angiofibromas, hypo-pigmented skin lesions on the lower back and dorsum of the right wrist, and previous history of seizures who was referred to the radiology department of the Korle Bu Teaching Hospital for Magnetic Resonance Imaging (MRI) of the brain. The MRI of the brain revealed subependymal giant cell astrocytomas, subependymal nodules, and cortical tubers. Ultrasonography of the abdomen also showed multiple angiomyolipomas and multiple simple cysts in both kidneys. The aim of this case report is to present the imaging findings and create awareness that this rare genetic disorder does exist in Ghana and advocate for formation of support groups for parents with children with tuberous sclerosis.

## Introduction

Tuberous sclerosis (TS), also known as epiloia, is a rare genetic disorder of autosomal-dominant inheritance with a prevalence ranging from one in 6,000 to one in 12,000. Both sexes and all ethnic groups can be affected [1-3]. It is a multisystem disorder involving the brain, skin, heart, kidneys, eyes, lungs, and liver which manifests only in late childhood [4]. Von Recklinghausen and later Desiree-Magloire Bourneville described this disorder in the 19th century and key pathological findings in some affected individuals were noted [5,6].

It is known that mutations on either of the two genes, TSC1 and TSC2, which encode for the proteins hamartin and tuberin, respectively, are the causes of TS. These proteins are tumor growth suppressors, which are agents that regulate cell proliferation and differentiation [7]. The classic triad of TS is seizures, mental retardation, and angiofibromas but this occurs in only 29% of patients with TS [8,9]. It is important to note that skin involvement is crucial for suspecting the diagnosis of TS [10,11].

We present the radiologic findings of a four-year-old boy with multiple facial angiofibromas who was referred to the radiology department of the Korle-Bu Teaching Hospital with a provisional diagnosis of TS.

## Case presentation

**Figure 1:**
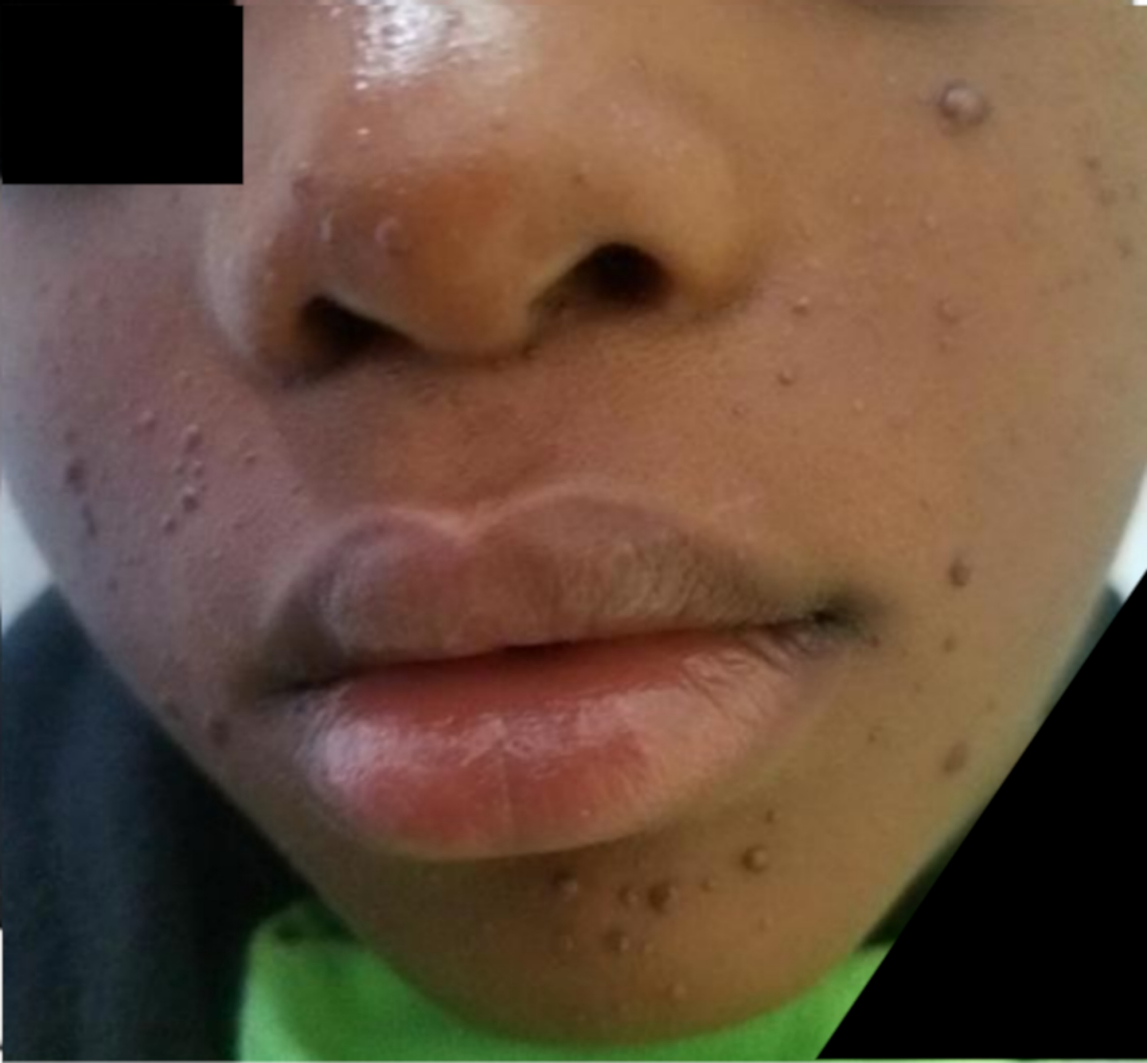
Multiple angiofibromas on the lower face.

**Figure 2:**
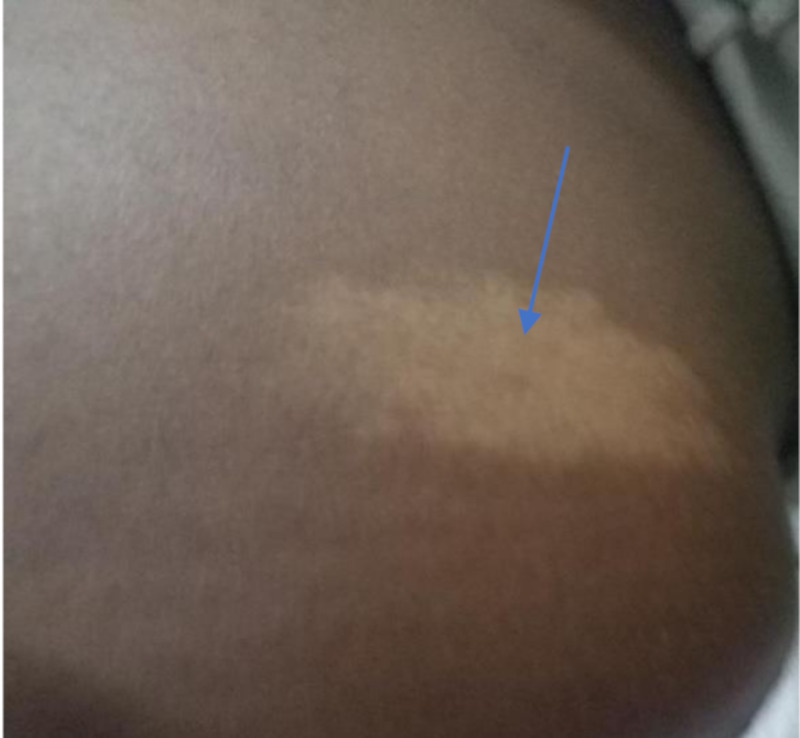
Hypopigmented macule (ash leaf spot) on his lower back close to his right flank/ lumbar region.

A four-year-old boy weighing 25kg was referred from the out-patient dermatology clinic of the Korle-Bu Teaching Hospital to the radiology department for brain MRI with a provisional diagnosis of TS. The physical examination in the radiology department revealed multiple skin lesions (angiofibromas) (Figure 1) on his lower face, which his mother said were appearing gradually since he was two years old and now had increased in number. We also noticed a hypopigmented skin lesion (ash leaf spot) (Figure 2) on his lower back close to his right flank. Mother admitted to the application of topical agents which were unsuccessful in clearing the lesions; she therefore took the child to the dermatology clinic. She also revealed that when her son was 2.5 years old, he was having multiple seizures which were generalized tonic-clonic in nature that lasted for a few minutes, about three times in a day for a period of four months and was treated at an outside hospital. She couldn’t remember the anticonvulsants given during that period. The boy no longer had seizures presently and is no longer on medication for seizures. He has normal cognitive function and does well at school; this was confirmed during our interaction with him prior to imaging. He was very active and communicated well for his age. There was no extended family history of this type of dermatological or seizure disorder. Pre-natal and post-natal periods were uneventful according to the mother. He had no delayed milestones. He is the second child of three siblings, all healthy and doing well.

**Figure 3:**
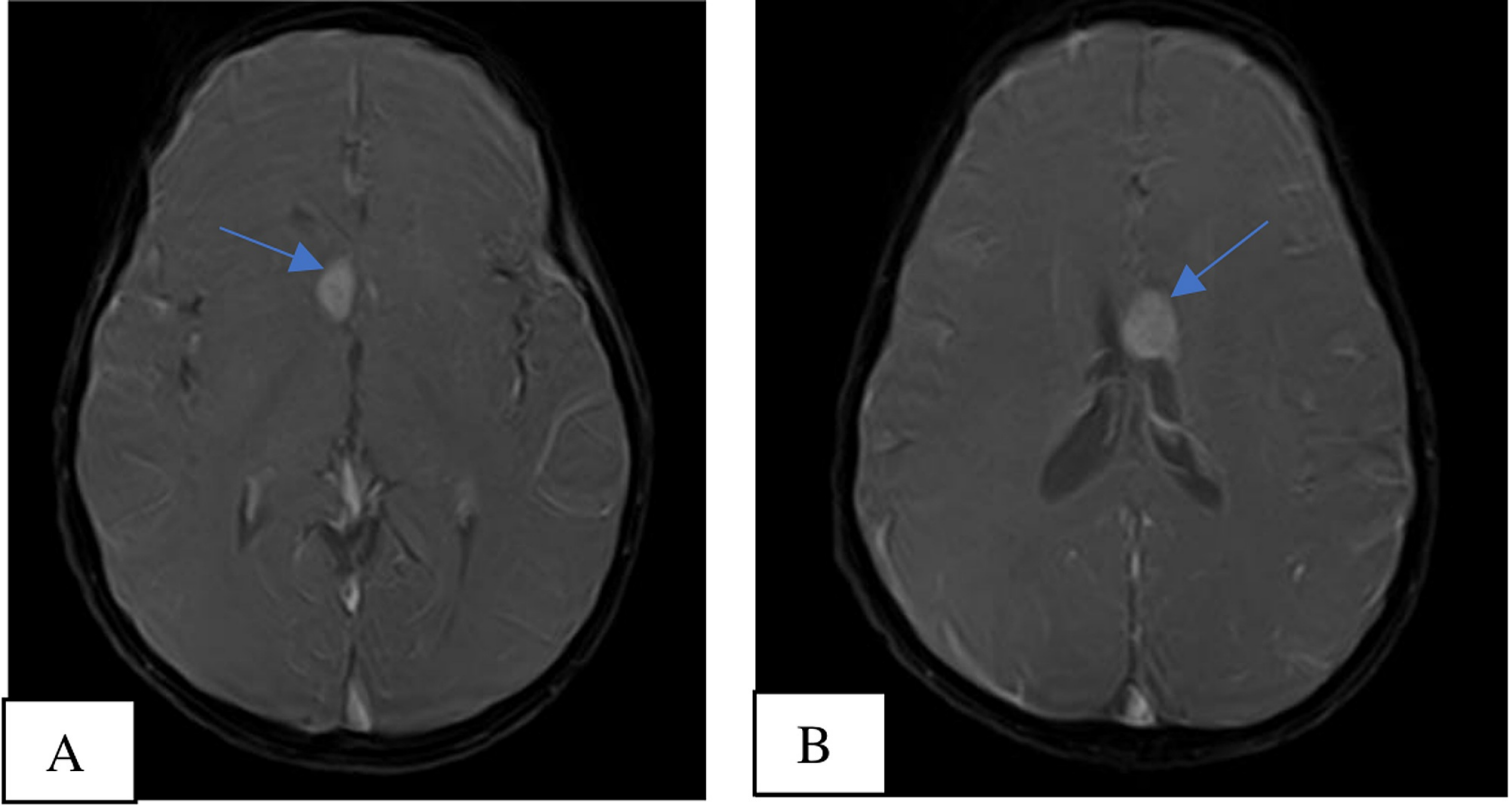
MRI, T1 axial FatSat contrast enhanced images at the level of the 3rd ventricle (3A) and body of the lateral ventricles (3B) showing the right and left Sub ependymal Giant Cell Astrocytomas (blue arrows).

**Figure 4:**
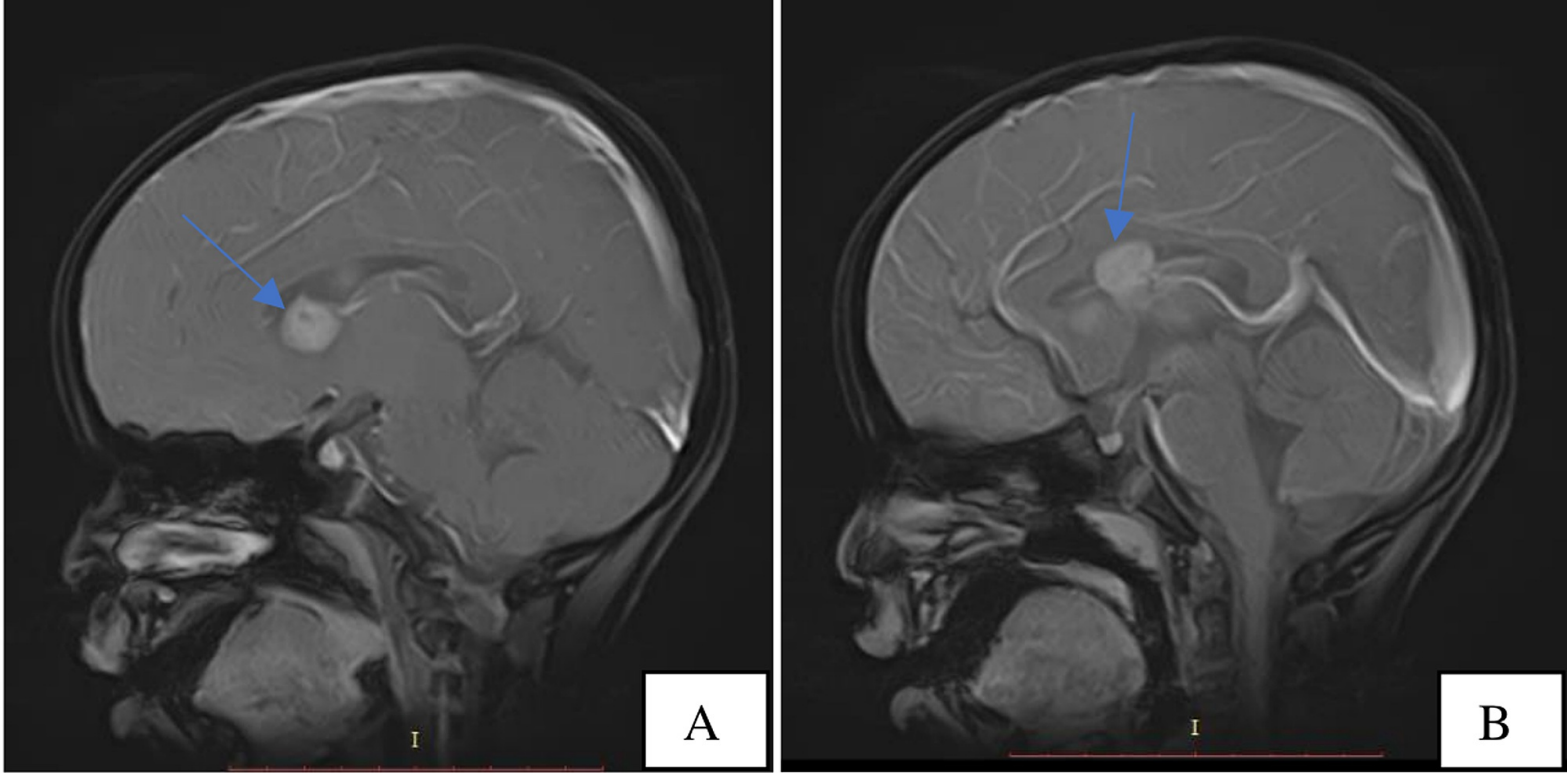
MRI, T1 Right para-sagittal (4A) and mid-sagittal FatSat contrast enhanced images (4B) showing the right & left Sub Ependymal Giant cell Astrocytomas approaching the Foramina of Monroe with no resultant hydrocephalus.

**Figure 5:**
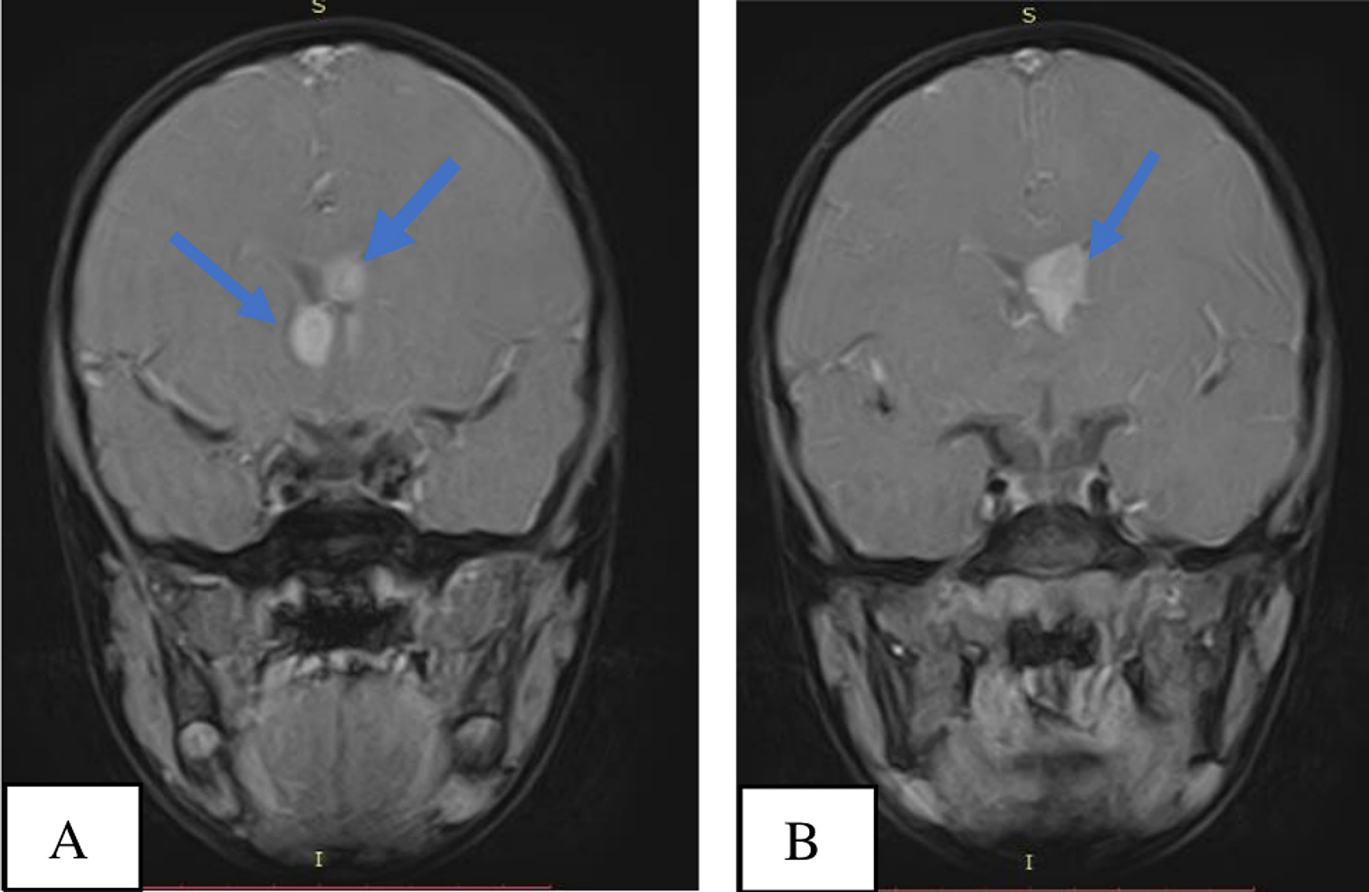
MRI , T1 Coronal FatSat contrast enhanced images showing the right & left Sub ependymal Giant Cell Astrocytomas (blue arrows) (5A, 5B) approaching the Foramina of Monroe . No resultant hydrocephalus.

**Figure 6:**
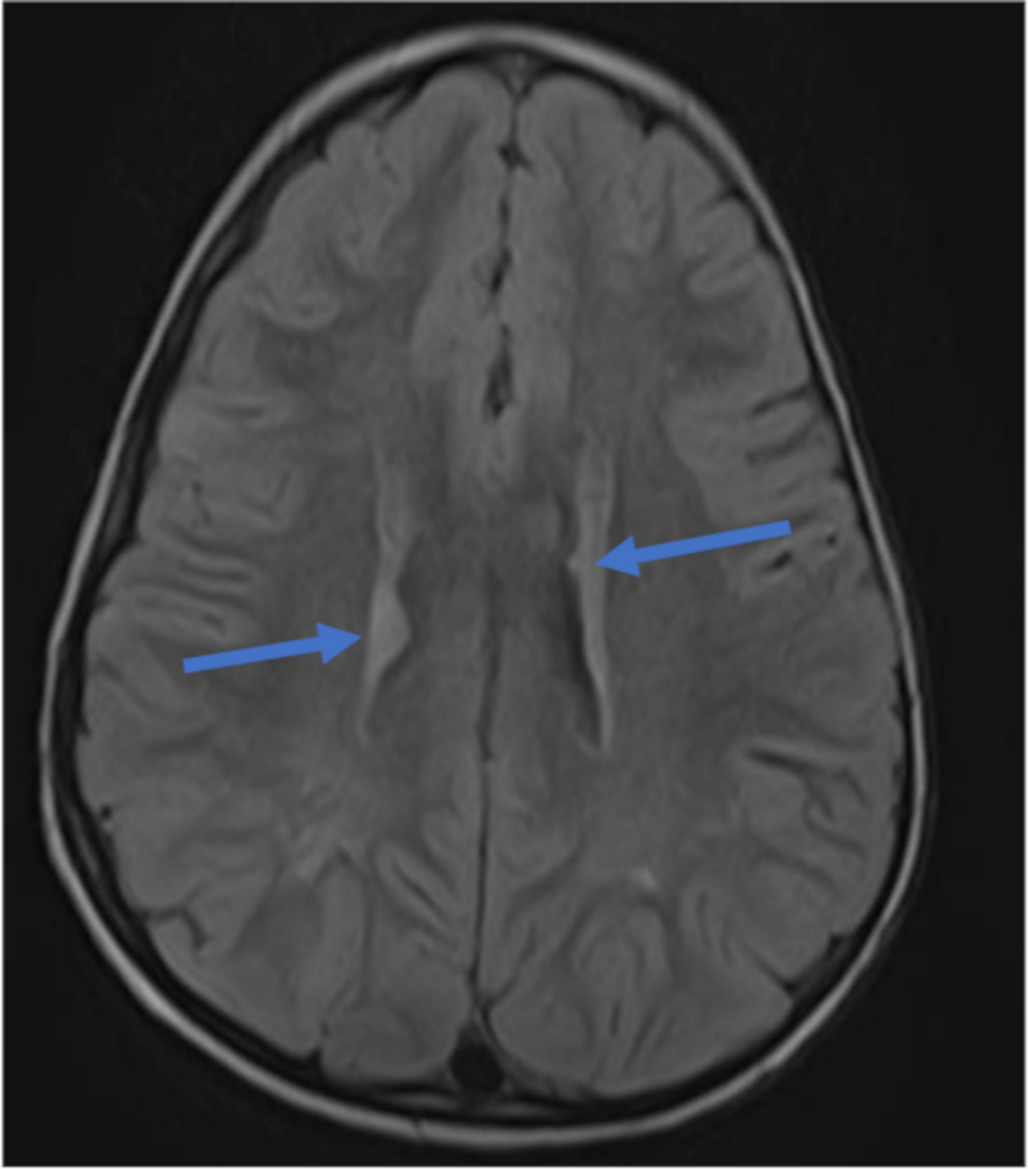
MRI, Axial FLAIR showing focal thickenings/nodules along the subependymal region of the body of the lateral ventricles, measuring 1.5mm in diameter representing tiny subependymal nodules (blue arrows).

After satisfactory assessment of his renal function and basic laboratory workup which included liver function tests, routine urine examination, complete blood count and hemoglobin, the following unenhanced brain MRI pulse sequences: T1-weighted (T1W) axial, T1 sagittal, fluid-attenuated inversion recovery (FLAIR) axial, T2-weighted (T2W) axial, diffusion-weighted imaging (DWI) axial, apparent diffusion coefficient (ADC), T2W axial FatSat and T1W axial FatSat were acquired using Toshiba Achieva 1.5 Tesla MRI Scanner under sedation. Intravenous gadolinium-based contrast medium (Magnevist) with a dose of 4mg/ml was administered and T1 FatSat sequence of the brain was repeated in the three orthogonal planes to assess enhancement patterns of the brain lesions that were visualized on the unenhanced images. The brain MRI showed two fairly defined, rounded, T1, T2 and FLAIR isointense (isointense to grey matter) subependymal masses approaching the right and left foramen of Monroe measuring approximately 1.5 x 1.1 x 0.8 cm and 1.6 x 1.5 x 1.1 cm, respectively, representing subependymal giant cell astrocytomas (Figures 3-5). The mass on the left extended minimally to the body of the lateral ventricle. They showed homogenous enhancement pattern. No associated obstructive hydrocephalus or ventriculomegaly was visualized. Focal thickenings/nodules are also seen along the subependymal region of the body of the lateral ventricles, measuring 1.5 mm in diameter suggesting tiny subependymal nodule (Figure 6).The rest of the cerebral parenchyma showed normal MR features and enhancement pattern; no obvious shift of midline structures. The third and fourth ventricles were of normal size.

**Figure 7:**
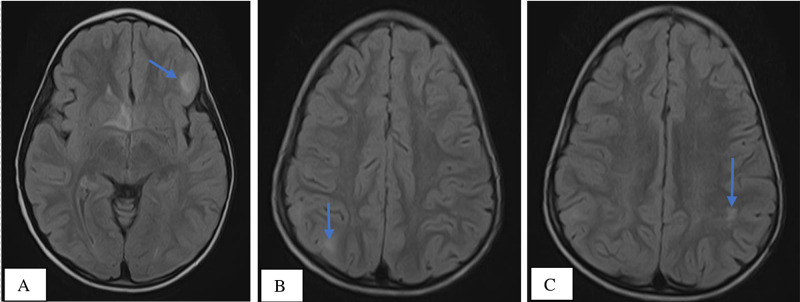
MRI, Axial FLAIR images showing well-defined ovoid hyperintense lesion measuring (1.3 x 0.7) cm in the subcortical region (grey-white matter interface) of the left frontal lobe (7A), a similar but smaller lesion-measuring (0.6 x 0.4) cm is seen in the subcortical region of the right parietal lobe (7B) and the left parietal lobe (7c) respectively all representing subcortical tubers (blue arrows).

A well-defined tiny, ovoid hyperintense lesion measuring 1.3 x 0.7 cm seen in the subcortical region of the left frontal lobe on FLAIR imaging and shows ‘restricted’ diffusion on DWI/ADC imaging in keeping with subcortical tuber. A similar but smaller lesion-measuring 0.6 x 0.4 cm is also seen in the cortex of the left parietal lobe and the right parietal lobes respectively all representing subcortical tubers. No obvious enhancement seen post contrast administration (Figure 7).

**Figure 8:**
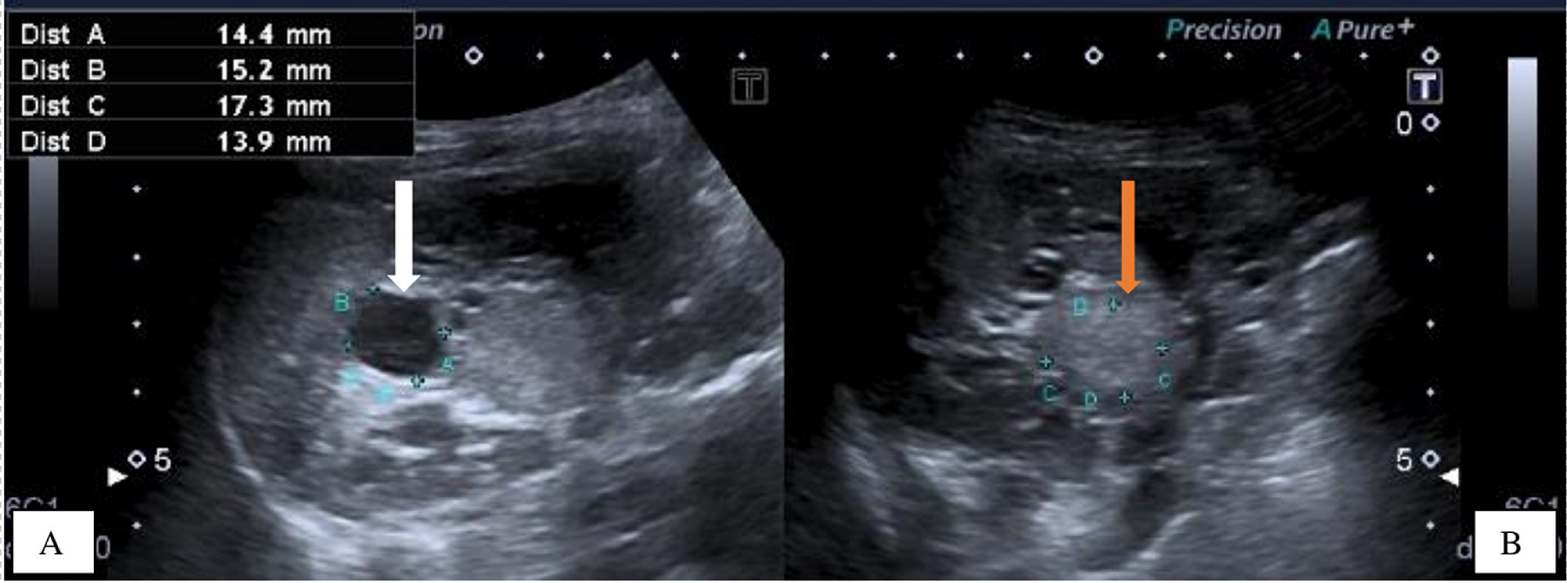
Ultrasound of the right kidney showing the simple renal cyst (white arrow) (8A) and the echogenic mass /angiomyolipoma (orange arrow) (8B) respectively.

**Figure 9:**
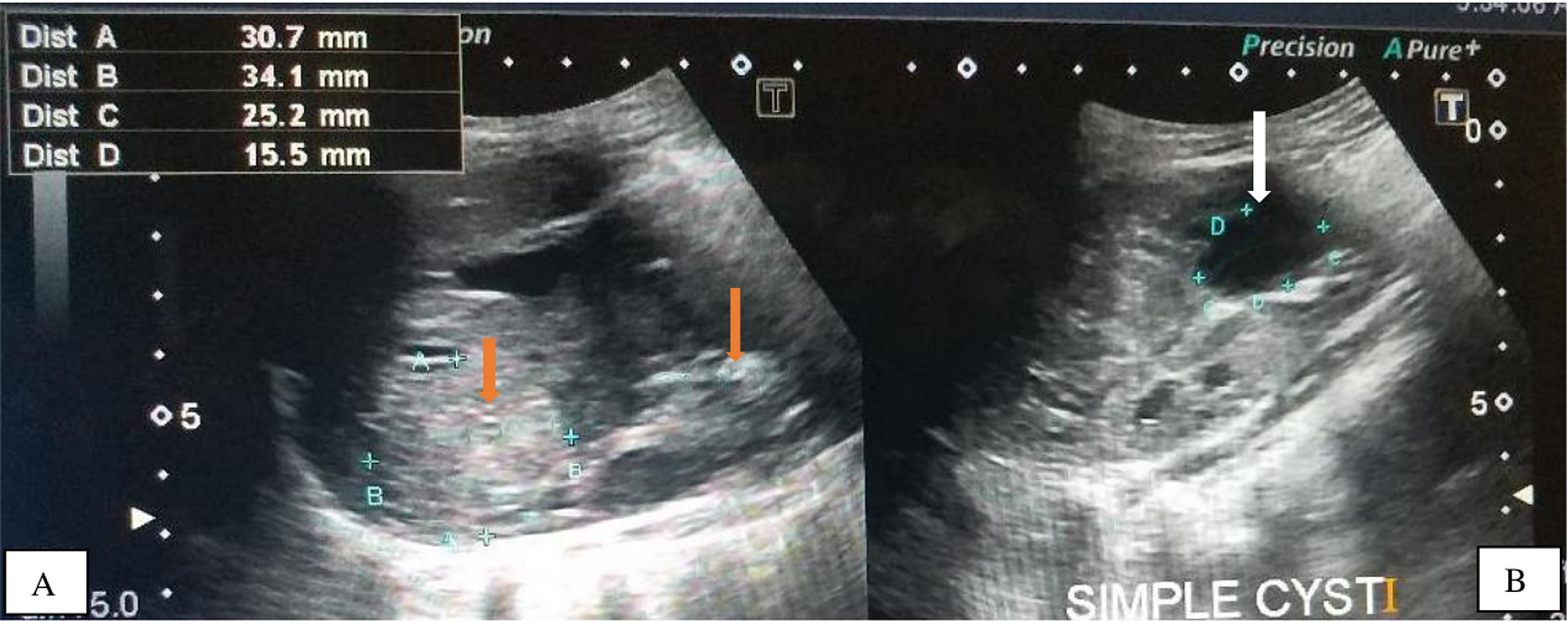
Ultrasound of the left Kidney showing the echogenic masses /angiomyolipoma (orange arrows) (9A) and the simple renal cyst (white arrow) (9B) respectively.

The clinical history, skin lesions and the brain MRI findings strongly suggested TS, hence we sought permission from the mother and performed an abdominal ultrasonography which revealed mildly enlarged right and left kidneys for age measuring 9.1 x 4.4 x 3.1 cm and 10.7 x 6.0 x 4.8 cm respectively, which have heterogeneous echopattern due to well-defined, rounded hyperechoic masses in both renal parenchyma representing angiomyolipomas. The largest two angiomyolipomas measured 1.7 x 1.4 cm and 0.5 x 0.4 cm on the right and 3.7 x 2.3 cm and 1.5 x 1.4 cm on the left. Also visualized were multiple tiny, simple renal cortical cysts, largest measured 1.5 x 1.4 cm on the right and 1.6 x 1.4 cm on the left. No hydronephrosis were seen bilaterally (Figures 8, 9).

An echocardiography was also done which was unremarkable. Chest radiograph was also normal.

Considering all our imaging findings which involved the brain, kidneys and including the cutaneous lesions which are the typical features of TS, we made a diagnosis of TS in our radiological report and the patient was referred to the dermatology clinic and pediatric neurologist [12,13]. Unfortunately the patient was lost to follow-up.

## Discussion

Tuberous sclerosis (TS) is a rare autosomal dominant inherited neurocutaneous syndrome that consists of hamartomatous lesions in the brain and kidneys, to mention just a few. This genetic disorder has no predilection for sex or ethnic group. The estimated prevalence ranges from one in 6,000 to one in 12,000 and approximately two-thirds of the cases are sporadic; this may explain why the patient did not have a family history of TS [1,2,14].

TS typically shows three clinical features, the Vogt triad which consists of mental retardation, epilepsy, and facial angiofibroma; however, approximately half of TS patients have normal intelligence and a quarter do not have seizures. Hence, it is not surprising that our patient had normal intelligence at this stage of the disease process and thus may have fallen into the proportion which has normal intelligence [8,9,12]. Mutations in the tumor-suppressor gene TSC1 (encoding hamartin) or, more commonly, TSC2 (encoding tuberin) are implicated in the pathogenesis of TS via a loss of inhibition of the mammalian target of rapamycin (mTOR) pathway. This subsequently leads to the growth of hamartomas in various organs, including the brain (cortical tubers, subependymal nodules, and subependymal giant cell astrocytomas), kidneys (renal angiomyolipomas), lungs (lymphangioleiomyomatosis), heart (cardiac rhabdomyomas), and skin (angiofibroma, Shagreen patch and hypomelanotic macule). The four-year-old boy in this case had a normal chest radiograph and the echocardiography was unremarkable. This is not surprising since the cardiac rhabdomyomas are usually seen in fetuses and neonates and disappear during infancy. Our patient had the classic brain findings on MRI which are subependymal giant cell astrocytomas, cortical tubers, and sub-ependymal nodules and the abdominal ultrasonography also showed the renal angiomyolipomas and renal cysts [9,15,16,17].

Major neurological manifestations of TS include seizures, autism, and behavioral and psychiatric disorders. Seizure is present in about 80-90% of patients which begin during the first year of life; varying from subtle focal seizures, infantile spasms, to generalized seizures. Our patient had seizures that seemed resolved but the typical brain lesions were evident as mentioned earlier, he however did not manifest any autistic or psychiatric disorder during our interaction with him at the time of imaging [2].

Cutaneous lesions in TS are hypomelanotic macule (90%), facial angiofibroma (75%), and Shagreen patch (20-30%) [3]. The hypomelanotic macules are seen at birth and almost all lesions are evident within the first two years of life. Facial angiofibromas are present during preschool years in the malar area as a spread of small pink or red spots across the cheeks and nose in a “butterfly distribution” and sparing the upper lip, this was the pattern in our patient (Figures 1, 2). Combining his past clinical history of seizures, the cutaneous manifestations, and the imaging findings, a definitive diagnosis of TS was made and this conforms to the Tuberous Sclerosis Complex Consensus which now requires two or more distinct types of lesions, rather than multiple lesions of the same type in the same organ system [13].

Patient was referred to the dermatologist and the pediatric neurologist for symptomatic treatment and counselling of the family but was lost to follow-up.

## Conclusions

TS is a neurocutaneous syndrome and it is a rare genetic disorder. In addition to the clinical history of seizures and cutaneous lesions, imaging plays an important role in diagnosis. The moment this is suspected the individual’s parents should be counselled and enrolled in multi-disciplinary treatment programs. It is true there is no cure but symptomatic treatment is available. Support groups should be formed in the country and individuals encouraged to join.
